# The Case for Endothelial Preservation *via* Pressure-Regulated Distension in the Preparation of Autologous Saphenous Vein Conduits in Cardiac and Peripheral Bypass Operations

**DOI:** 10.3389/fsurg.2016.00054

**Published:** 2016-09-22

**Authors:** Eric S. Wise, Colleen M. Brophy

**Affiliations:** ^1^Department of Surgery, University of Maryland Medical Center, Baltimore, MD, USA; ^2^VA Tennessee Valley Healthcare System, Vanderbilt University, Nashville, TN, USA; ^3^Department of Surgery, Vanderbilt University Medical Center, Nashville, TN, USA

**Keywords:** vein graft preparation, endothelium, vascular, saphenous vein graft, CABG, bypass

## Introduction

The human saphenous vein (HSV) remains the most common conduit for peripheral and aortocoronary bypass operations in the United States (1, 2), with ever improving, though suboptimal long-term patency rates. The mishandling of vein grafts during explantation, preparation, and autotransplantation is gaining attention as a source of injury portending increased short-term thrombosis as well as acceleration of neointimal formation, the most common cause of vein graft failure from 2 months to 2 years postoperatively (3, 4). The handling of vein grafts during the explantation has been thoughtfully addressed, as “no-touch” open harvest has emerged as an approach stressing a minimization of trauma to tissue handling. In “no-touch” harvesting, the graft is removed with a pedicle of surrounding tissue. This has been shown to maintain structural and functional integrity of all cell layers, prevent spasm (5), and improve graft patency (6, 7), while conferring protection from subsequent intraoperative manipulations (8, 9).

Nonetheless, once HSV has been fully explanted, manipulations to the conduit are routinely performed to make the anastomosis technically simpler and safer. The most well-characterized and injurious of these intraoperative manipulations is intraluminal radial distension (10). HSV is routinely cannulated and distended using a handheld syringe to infuse a solution or autologous blood, which dilates the vein. This procedure serves to disrupt any valves, increase the luminal diameter, identify leaks or injuries, prevent spasm, and ultimately, facilitate an easier anastomosis (11, 12). It is the opinion of the authors that this practice should be done with great care, emphasizing minimization of distension pressure using either controlled distension or a tool, such as a pressure release valve. While not conclusive, existing data strongly suggest that mitigation of distension-induced graft injury may prevent both early and delayed graft failure due to thrombosis and neointimal hyperplasia, respectively.

## Summary of the Literature

Starting in the late 1970s, the practice of unregulated intraluminal distension became the target of significant research (13, 14). Biochemical and biomechanical effects of intraoperative distension were progressively characterized throughout the next several decades. Unregulated distension of HSV graft can exert radial pressures as great as 600–700 mmHg, far in excess of physiologic systolic blood pressure, which of itself is supraphysiologic to the unadapted venous wall (11, 13, 15, 16). There are myriad biochemical and biomechanical changes to the cells and extracellular matrix that occur in HSV conduit upon arterial implantation, with chronic exposure to an arterial distension pressure. However, there is a unique profile of biochemical and biomechanical changes that have been shown to occur with acute intraoperative distension, particularly involving the critical endothelial layer whose integrity is most indispensable for graft patency.

Intraoperative distension exerts a brief unidirectional force similar to laminar shear stress on the conduit. The harmful biochemical and biomechanical effects induced are decidedly different from those observed in chronically arterialized endothelial cells (14). Chiefly, distension of HSV leads to significant and pressure-dependent endothelial denudation. This was first reported in 1980, when scanning electron microscopy revealed qualitatively severe endothelial damage in monkey saphenous vein at 700 mmHg distension, though normal morphology was observed at pressures of 300–400 mmHg (13). While several ensuing studies over the decade demonstrated a qualitative destruction of the endothelial monolayer, Angelini and colleagues first quantified this by using stimulated prostacyclin generation of vascular tissue as a surrogate for the presence of an endothelial cell. In their formative experiment, concentrations of prostacyclin were measured in fresh and surgically prepared HSV, both spontaneously and after vortex stimulation. Prostacyclin is an endothelial-derived prostaglandin, which works synergistically with nitric oxide to prevent thrombosis (17, 18). Angelini et al.’s findings that vortex-stimulated prostacyclin generation increased in response to surgical preparation were expanded to determining that it was indeed the distension aspect of surgical preparation, particularly with pressures greater than 300 mmHg, that most contributed to impaired stimulated prostacyclin generation (19). Measuring stimulated prostacyclin as a surrogate marker, this allowed the reliable quantification of degree of de-endothelialization that would become essential in multiple future experiments (19).

Microscopic quantification of denudation has further been quantified in several modern reports. Chester and colleagues reported a significant decrement in staining for endothelial markers endothelial nitric oxide synthase (eNOS), platelet endothelial cell adhesion molecule (CD31), and von Willebrand Factor (vWF) in segments of HSV distended to 300 mmHg, though no changes were observed at 100 mmHg distension relative to non-distended control tissue (20). Also, 300 mmHg was a critical distension pressure for decreased expression of the endothelial marker CD34 on HSV (21, 22). Similar results have been reported demonstrating impaired endothelial staining after full surgical manipulation, though surgical skin marking or preservation in solution could contribute to these findings (12, 23–26). Seminal work by Stigler and colleagues revealed sequentially increasing HSV endothelial denudation from median loss of 29–54% to 75–91% at respective pressures of 50, 100, 150, and 300 mmHg, as determined by CD31 staining, though HSV grafts were exposed to the pressure continuously for 30 min (27). In a porcine model, unregulated distension of saphenous vein was recently been shown to cause significantly decreased eNOS and CD31 staining, changes fully mitigated by distension *via* an in-line pressure release valve that limits intraluminal pressure to a more physiologic 140 mmHg (11).

Functional assessment of vascular endothelium is easily quantified using an organ bath. The apparatus allows for the suspension of vascular smooth muscle tissue on a force transducer (28). The addition of acetylcholine or a cholinomimetic to a submerged vascular ring can cause activation of eNOS with subsequent nitric oxide generation and release. Nitric oxide then causes a cGMP-mediated vascular smooth muscle relaxation, the degree of which can be quantified. Endothelial-dependent physiologic responses are a valuable surrogate for denudation; however, there are limitations to its interpretation. Endothelial responses may be decreased in part due of endothelial cells that are dysfunctional in their production and release of nitric oxide. Data suggesting a significant component of endothelial dysfunction were reported by Okon and colleagues in 2004; vWF staining on distended veins remained intense, despite loss of endothelial-dependent relaxation (29). Additionally, distension is known to cause mild smooth muscle necrosis, decreasing the graft’s responsiveness to endothelial-derived nitric oxide (30, 31).

Distension has shown the ability to upregulate markers of inflammation that are governed by the activation of nuclear factor-κB (NF-κB) and subsequent generation of the cytokine TNF-alpha (24, 32–34). HSV distended at 300 mmHg for 2 min demonstrated an average of 33% endothelial denudation; however, significant upregulation of endothelial intercellular adhesion molecule (ICAM-1), vascular cell adhesion molecule (VCAM-1), and P-selectin was also observed (35). In addition to NF-κB activation, lack of nitric oxide generation may also contribute to this change (35–37). Upregulation of these cell surface adhesion molecules promotes interactions with integrins on circulating neutrophils, monocytes, and platelets with subsequent transmigration into the vessel wall, events known to be early steps in graft obstruction (35). P-selectin, in particular, is implicated in the promotion of platelet aggregation in regions of vascular injury, leading to thrombosis (38). Its downregulation in vascular endothelium represents an emerging approach in the treatment of vein graft failure (38). Additionally, increased vascular permeability has been linked to ICAM-1 expression (33, 39).

These findings were replicated by Khaleel and colleagues in 2012 (16). Additional endothelial markers of inflammation were also found to be upregulated as well, *via* immunostaining and real-time polymerase chain reaction analyses. Toll-like receptors TLR2 and TLR4 were both increased in distended HSV, in direct proportion to luminal distension pressures. The expression of these receptors too is enhanced by TNF-alpha and contributes to immune system activation in cases of pathogen-induced inflammation (40). Their expression may accelerate vein graft failure as well. Recent data suggest that TLR2 expression may promote graft failure in vascular tissue and that this may be due to its promotion of medial smooth muscle cell migration, a key event in atherogenesis and neointimal formation (40–42). A marker of acute inflammation, endothelial TLR4 upregulation was also seen in distended HSV (16, 40). Its contribution to neointimal formation is well-established, as TLR4-deficient mice have shown not to develop a neointima (16). However, these consequences are primarily due to TLR4 upregulation on vascular smooth muscle cells rather than endothelium (43). Furthermore, both endothelial TLR2 and TLR4 have been implicated in the binding of the β_2_-glycoprotein moiety on circulating inflammatory cells, promoting thrombosis (44, 45).

While penetration of circulating monocytes into the vascular medial layer represents one of the inciting events of atherogenesis, subendothelial migration with lipid accumulation and formation of foam cells directly follows (46). Distension leads to increased expression of endothelial scavenger receptors SR-A and SR-B (16). Largely found on macrophages, these receptors are also found in vascular smooth muscle cells and endothelium. Ligand binding to these receptors amplified its expression and generates a positive feedback loop. Consequences of distension-induced expression and ligand binding include lipid peroxidation, endothelial dysfunction, apoptosis, and most concerningly, foam cell formation.

## Conclusion

While only a few key offenders are summarized herein, there are a host of other adhesion molecules, toll-like receptors, and scavenger receptors that are ostensibly overexpressed in distended HSV, particularly those that are also regulated by NF-κB translocation (24). Many of these changes likely contribute to vein graft failure. There are no studies in human patients examining the influence of vein graft distension pressure on patency; however, in the authors’ opinion, minimization of distension represents a simple modification to the conduit preparation process that may very well prevent graft failure, the extent to which, however, is unclear. Options exist to minimize distension, however, including pressure-regulated syringes or pressure release valves to prevent undue pressurization of the saphenous vein grafts (25, 26). These represent simple modifications to the vein graft preparation process that preserve functional and structural properties of the conduit endothelium, and thus, may very plausibly abrogate or retard thrombosis and neointimal hyperplasia.

## Author Contributions

EW contributed to research, writing, and critical revision of the manuscript. CB oversaw all aspects of the research and composition of the manuscript. All authors gave final approval.

## Conflict of Interest Statement

CB has a proprietary interest in VasoPrep Surgical, Inc. EW has no conflicts of interest to disclose.
